# Nanobodies for Medical Imaging: About Ready for Prime Time?

**DOI:** 10.3390/biom11050637

**Published:** 2021-04-26

**Authors:** Léa Berland, Lauren Kim, Omar Abousaway, Andrea Mines, Shruti Mishra, Louise Clark, Paul Hofman, Mohammad Rashidian

**Affiliations:** 1 Department of Cancer Immunology and Virology, Dana-Farber Cancer Institute, Boston, MA 02215, USA; lea_berland@dfci.harvard.edu (L.B.); lkim@college.harvard.edu (L.K.); omarb_abousaway@dfci.harvard.edu (O.A.); amines@bu.edu (A.M.); shruti_mishra@dfci.harvard.edu (S.M.); louisem_clark@dfci.harvard.edu (L.C.); 2 Université Côte d’Azur, CNRS, INSERM, IRCAN, 06100 Nice, France; hofman.p@hu-nice.fr; 3 Department of Chemistry and Bioengineering, Harvard University, Cambridge, MA 02138, USA; 4 Laboratory of Clinical and Experimental Pathology, FHU OncoAge, Nice Center Hospital, 06100 Nice, France; 5 Department of Radiology, Harvard Medical School, Boston, MA 02115, USA

**Keywords:** nanobodies, VHHs, molecular imaging, radionuclide imaging, immuno-PET, SPECT/CT, cancer-specific markers, immune checkpoint imaging

## Abstract

Recent advances in medical treatments have been revolutionary in shaping the management and treatment landscape of patients, notably cancer patients. Over the last decade, patients with diverse forms of locally advanced or metastatic cancer, such as melanoma, lung cancers, and many blood-borne malignancies, have seen their life expectancies increasing significantly. Notwithstanding these encouraging results, the present-day struggle with these treatments concerns patients who remain largely unresponsive, as well as those who experience severely toxic side effects. Gaining deeper insight into the cellular and molecular mechanisms underlying these variable responses will bring us closer to developing more effective therapeutics. To assess these mechanisms, non-invasive imaging techniques provide valuable whole-body information with precise targeting. An example of such is immuno-PET (Positron Emission Tomography), which employs radiolabeled antibodies to detect specific molecules of interest. Nanobodies, as the smallest derived antibody fragments, boast ideal characteristics for this purpose and have thus been used extensively in preclinical models and, more recently, in clinical early-stage studies as well. Their merit stems from their high affinity and specificity towards a target, among other factors. Furthermore, their small size (~14 kDa) allows them to easily disperse through the bloodstream and reach tissues in a reliable and uniform manner. In this review, we will discuss the powerful imaging potential of nanobodies, primarily through the lens of imaging malignant tumors but also touching upon their capability to image a broader variety of nonmalignant diseases.

## 1. Introduction to Molecular Imaging

With regards to oncologic management, imaging is heavily relied upon for early diagnosis and follow-up assessment of treatment response. Current recommendations frequently involve response evaluation criteria in solid tumors (RECIST) [1], which utilizes the reproducible structural imaging methods of Magnetic Resonance Imaging (MRI) and Computer Tomography (CT) scans to computationally measure the evolution of tumor size. However, these techniques alone do not image biological processes within the body and thus cannot provide information at the cellular or molecular levels. Complimentary to structural imaging, tissue biopsies followed by histological assessment have long remained the gold standard for cancer-specific diagnostics. However, tissue biopsies are invasive and often associated with sampling pitfalls, notably due to tumor heterogeneity. This makes them unideal for fully assessing the response to targeted or immune-modulating treatments.

In filling this niche, molecular imaging has developed as a technique to become a crucial part of modern medicine. The ability to visualize, characterize, and monitor biological processes facilitates the analysis of each individual patient’s unique molecular patterns, thus making it a pivotal component of precision medicine [2]. The field utilizes a variety of tools, such as small molecules [3], peptides [4], proteins [5], antibodies and antibody fragments, and nanoparticles [6], to identify targets specific to the biological or cellular processes of interest. While also important in early disease stages, the ability to personalize treatment approaches is especially crucial in the later stages of oncologic, cardiovascular, and neurologic disease, when patient quality of life is often a primary concern in deciding whether to administer therapeutics, especially those with undetermined efficacy [7,8,9].

Noninvasive imaging approaches, such as positron emission tomography (PET), single-photon emission computed tomography (SPECT), and targeted contrast-enhanced MRI, enable visualization of specific events and target cells within the body {Citation}. While methods involving MRI imaging have been used extensively in the past, the scope of such strategies is limited because they fail to provide quantitative information. PET and SPECT imaging techniques, which are covered in greater detail throughout this review, can help quantify biological processes and are thus highly useful alternatives for target-molecule and target-cell visualization. Furthermore, hybrid imaging techniques, such as PET/CT, SPECT/CT, or PET/MRI, provide dynamic, whole-body images that combine both structural and functional information in a singular scan [7,8]. As such, these multimodal imaging methods have potentiated molecular imaging and are now widely used as an adjunct to tissue biopsies [10].

Fluorine-18 (^18^F) is a commonly used PET radioisotope with a short half-life (t_1/2_ of ~110 min) and high specific activity [9]. Small molecules, such as fluorodeoxyglucose [^18^F]FDG and fluorothymidine [^18^F]FLT, are commonly used PET tracers [11,12,13]. Metabolically active, glucose-avid cells, such as fast-proliferating cancer cells and, to some extent, activated immune cells, demonstrate high uptake of the administered [^18^F]FDG radiotracer. Thus, FDG-PET imaging has proven to be a very useful tool in the detection of metastatic lesions. However, [^18^F]FDG is non-specific; its absorption occurs in all cells expressing GLUT1/GLUT3 transporters, including those associated with inflammation, which leads to a radiotracer uptake comparable to that of metastatic cells [14,15,16,17]. Furthermore, false-negative results can occur in certain cancer types, such as those that use alternative metabolic pathways, with downregulation of the GLUT1/GLUT3 channels [18]. Similarly, [^18^F]FLT, commonly used to observe Thymidine Kinase 1 (TK1) activity, cannot be used specifically for immune cell imaging as other cancer cells with noticeable TK1 activity also demonstrate indistinguishable uptake [19,20]. This inherent lack of target specificity makes such small molecule tracers unsuitable in assessing immune response or imaging the dynamics of specific markers in response to treatment. To overcome these issues, molecules with high target specificity, such as antibodies, have been studied, radiolabeled, and used for said imaging purposes.

## 2. Use of Antibodies for Targeted Imaging

**Figure 1:**
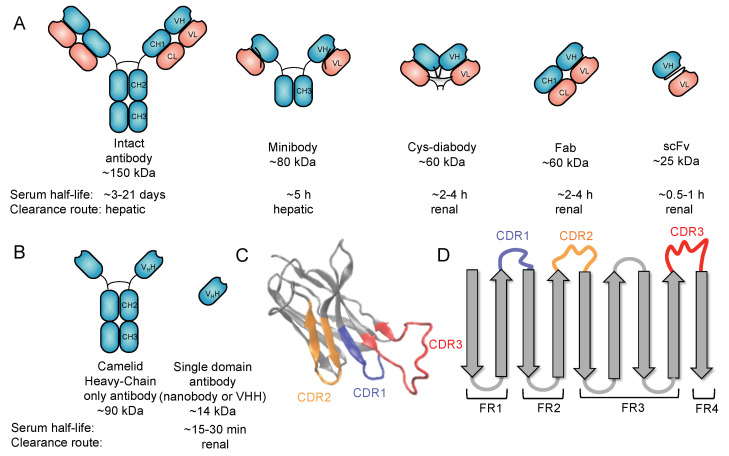
Human and camelid antibody fragments. (**A**) Structures of the human antibody fragments used for molecular imaging. Heavy chain is colored in blue and light chain in red. Antibody domains are labelled with their appropriate names (CH—constant heavy; VH—variable heavy; VL—variable light; VHH—variable heavy of heavy-chain-only antibodies). Size and circulation half-lives are mentioned below the structures. (**B**) Camelid heavy chain-only antibody and VHH antibody fragment. (**C**) Crystal structure of the nanobody (PDB ID: 6OS1) with residues of CDRs colored. (**D**) Schematic representation of the VHH domain framework and CDR regions. Arrows represent beta sheets.

Heterogeneity amongst the molecular phenotypes from the primary tumor to secondary lesions raises issues in the therapeutic management of many patients with advanced cancer. A patient rarely presents with a single metastatic lesion, and the presence of these scattered lesions significantly complicates biopsy procedures. As some lesions are located in hidden or inaccessible regions, it is often neither feasible nor practical to analyze all of them using traditional biopsies [21]. Alternatively, a liquid biopsy can be realized for molecular biology assessment, but it is common for many discrepancies to exist between the molecular profiles of tissue and liquid biopsies [22]. Metastatic disease of the vital organs is often identified as the principal cause of a high mortality rate in patients suffering from lung, colon, or breast cancer [23]. Thus, it is of vital importance to select a targeting agent that is able to track compatible molecular markers in both the primary and metastatic sites of the disease [24]. Radiolabeled antibodies, due to their high specificity, are often viewed as the optimal imaging agent of choice to address this issue. This approach is referred to as “immuno-PET”. Employing radiolabeled full-sized antibodies (~150 kDa) themselves as therapeutic agents is advantageous due to their ability to reveal treatment pharmacokinetics and pharmacodynamics [25]. In some cases, however, these full-sized antibodies are unable to efficiently penetrate the entirety of the tumor, leading to an incomplete visualization of the lesion [26]. Furthermore, their long circulatory half-lives (days to weeks) will not allow same-day imaging. To address these drawbacks, alternative antibody formats, such as minibodies (~80 kDa), diabodies (~60 kDa), and single-chain Fv fragments (scFvs, ~25 kDa) as imaging agents, have been developed (Figure 1A). Small protein scaffolds, such as affibodies (~7 kDa) [27], fibronectins (~10 kDa) [28,29], and DARPins (~14–18 kDa) [30,31], have been developed as well. These altered antibody formats and protein scaffolds have been found to require considerable engineering effort to achieve acceptable expression levels, circulatory half-lives, and stability. Attractive alternatives are single-domain antibody fragments, referred to as VHHs, or nanobodies (Figure 1B–D).

## 3. Nanobodies

In addition to conventional antibodies, camelids, such as llamas and alpacas, have unique heavy-chain-only antibodies [32]. These antibodies are unique in that the variable regions are encompassed by a single domain (VHH) instead of two separate domains (VH and VL) as seen in conventional antibodies [33]. The variable domains of the camelid heavy-chain-only antibodies have found widespread applications in biomedical research.

**Figure 2:**
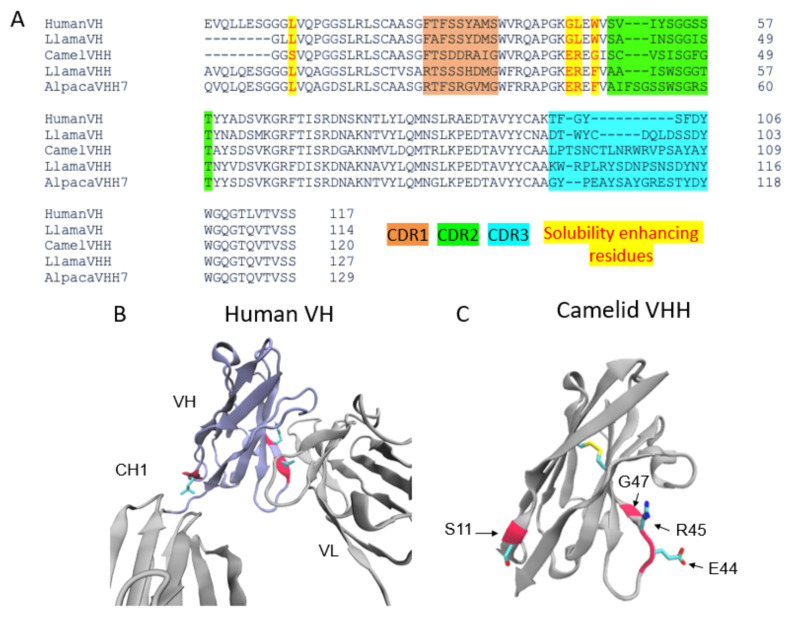
Architecture of VHHs promotes their solubility and stability. (**A**) Multiple sequence alignment of the Human VH, Llama VH, Llama VHH, Camel VHH, and Alpaca VHH domains. CDR regions are highlighted orange (CDR1), green (CDR2), and blue (CDR3). The VHH domains shown have a larger CDR3 region than the VH CDR3s. Highlighted in yellow and written in red are residues mutated to enhance solubility in VHH domains. Camel VHH bear four mutations (L11S, G44E, L45R, and W47G). Llama and alpaca VHHs bear G44E, L45R, as well as a W47F mutation. (**B**) Crystal structure (PDB ID 1IGY) of a human antibody with the VH domain colored in purple. Residues mutated in the camelid VHH domains are colored red. L11 makes contact with the CH1 domain while L45 and W47 form contacts with the VL domain. (**C**) Crystal structure of a camelid (camel) VHH (PDB ID 6U14). E44, R45, and G47 in the hypothetical VL binding site promote stability and solubility of the VHH as a single domain.

Nanobodies are highly water-soluble and stable, have high specificity, and can bind to their targets with high affinity, often in the low nanomolar range [34]. VHHs are stable as single-domain antibodies because of several mutations on their surface that allow them to be water soluble [34]. In particular, several residues that would be at the VH–VL interface in conventional antibodies are mutated for hydrophobic to hydrophilic residues (G44E, L45R, and W47G) (Figure 2), enhancing their stability and solubility as a single domain. In addition, there is a solubility enhancing mutation, most commonly found in camel VHHs, at the VH–CH1 interface (L11S) (Figure 2A,C).

The factor contributing to the high affinity of these nanobodies is that their frameworks have three complementarity-determining regions (CDRs). These CDRs are analogous to those found in human antibody VH and VL domains and are subject to somatic hypermutation in the course of affinity maturation. The CDR3 of VHHs is especially long in comparison to the human counterpart [35]. The length and flexibility of VHH CDR3s enable the nanobody to access a variety of conformations. In some cases, VHH CDR3s are able to fold back and make contact with the nanobody framework [35]. Taken together, these factors compensate for the lack of sequence variability incurred by the loss of VL CDRs, allowing VHHs to bind to their targets with high specificity and affinity (Figure 1C,D and Figure 2A).

**Figure 3:**
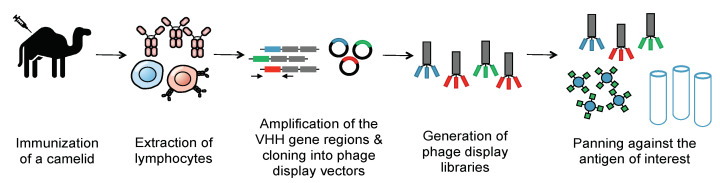
Generation of a nanobody library. To create an immune library, camelids are immunized against a molecule of interest. mRNA of the camelids’ peripheral blood mononuclear cells is then converted into cDNA. PCR is then employed to amplify the VHH genes. These immune VHH genes will then be cloned into a phage display vector. Phages are then generated using *E. coli* strains such as TG1. Phage libraries are then panned against immobilized antigens to select for nanobodies that selectively bind the antigen with high affinity. The panned libraries are then used for reinfection of *E. coli* to obtain specific clones.

Methods of generating nanobodies against an antigen of interest have already been well established [33]. In brief, a llama or alpaca (among a variety of other camelids) is immunized against the antigen(s) of interest [33]. Administration of the protein antigen is typically accompanied by an immune adjuvant that serves to enhance the overall immune response [36]. Several weeks later, blood is harvested from the immunized animal and peripheral blood mononuclear cells (PBMCs) are purified. This purification is then followed by total RNA extraction, VHH amplification, and finally, the construction of a phage display library. Phage display libraries are among the most common methods of preparing nanobody libraries, but other methods, such as *E. coli* or yeast display, could alternatively be used [33,37,38]. Finally, the lead VHHs are identified and expressed as soluble proteins using reliable approaches, such as magnetic activated cell sorting (MACS), fluorescence activated cell sorting (FACS), or panning against immobilized antigens (Figure 3) [39,40,41].

The short circulatory half-life of nanobodies have allowed the use of a range of isotopes with short half-lives for imaging, such as Galium-68 (^68^Ga, t_1/2_ = 67.71 min) and ^18^F (t_1/2_ = 109.7 min), as well as longer-lived isotopes, such as Technetium-99m (^99m^Tc t_1/2_ = 6.0 h), Copper-64 (^64^Cu t_1/2_ = 12.7 h), Indium-111 (^111^In t_1/2_ = 67.2 h), Zirconium-89 (^89^Zr t_1/2_ = 78.41 h), and Lutetium-177 (^177^Lu t_1/2_ = 6.7 days). Similar to other antibody fragments, nanobodies are commonly labeled nonspecifically via their side-chain lysine residues using chelators or radioisotopes that are functionalized with amine-reactive groups such as *N*-hydroxysuccinimide (NHS) or isothiocyanatobenzyl (pSCN) groups. While this strategy is robust and reproducible, it is not site-specific, which may damage antigen-binding sites [42]. To address this issue and to ensure the binding capacity is not compromised, a variety of site-specific labeling approaches, such as the use of sortase technology, have been developed [43]. Another common approach is using a His6 tag to install ^99m^Tc, a commonly used SPECT isotope [44].

## 4. Nanobodies as a Tool for Imaging Cancer

### 4.1. Imaging Cancer Cell Markers

#### 4.1.1. EGFR

The epidermal growth factor receptor (EGFR) is known to be overexpressed in several cancer types, such as lung adenocarcinoma, head and neck squamous cell carcinoma, and colorectal carcinoma [45,46]. In past years, treatments targeting this receptor have been developed, approved, and their efficacy demonstrated [47]. Imaging EGFR expression may provide valuable information to determine the appropriate treatment plan for a given patient. An anti-EGFR nanobody has been developed and radiolabeled to detect EGFR-positive tumors in animals [47,48]. SPECT images revealed that the ^99m^Tc-labeled nanobodies could specifically recognize tumor cells expressing EGFR, distinguishing them from non-tumor cells and minimizing falsity in the results [47]. The blood clearance rate of these nanobodies was rapid, with adequate visualization achieved merely 1.5 h post-injection.

Empirical results have gone on to confirm the high binding specificity and selectivity of the anti-EGFR nanobody, with a demonstrated difference in uptake between tumor cells overexpressing EGFR (A431 cells) and those with a slightly more moderate expression (DU145 cells) [47,48]. Concentration-dependent reductions in both cell viability and tumor uptake were observed when this model was treated with Erlotinib, a molecular agent targeting the tyrosine-kinase domain of the EGFR, which further reinforces the nanobody’s promisingly high affinity and receptor specificity [48]. This, along with its favorable biodistribution patterns, encourages further investigation for the nanobody’s translation into the clinic, as this could potentially provide insight into the implications of treatments targeting the same EGFR receptor.

#### 4.1.2. HER2

Breast cancer can be classified into four categories based on the expression of different cell surface proteins: estrogen-positive, progesterone-positive, human epidermal growth factor receptor 2 (HER2)-positive, and triple negative (void of all surface protein expression) [49]. The HER2 protein has been noted for its ability to induce rapid cellular growth and proliferation. Cancer cells expressing abnormally high levels of the HER2 proto-oncogene are thus often correlated with worse prognoses [50]. Cancer treatments designed to specifically target HER2 have already been proven to demonstrate wide efficacy and promising results [51,52]. Monoclonal antibodies against HER2 are now used to treat all stages of HER2/neu-positive breast cancer, with Trastuzumab being one of the most effective and commonly used types [53].

HER2-targeted imaging can provide valuable insight into the aggressiveness of the tumor and thus help in shaping the management and outcome of the treatment [50]. In a study, breast cancer patients were imaged with ^89^Zr radiolabeled Trastuzumab. Patients identified with HER2-negative primary tumors, but HER2-positive metastases further benefited from Trastuzumab treatment [54]. Therefore, immuno-PET will be a useful complementary technique to histology and immunostaining of such cancers. Nanobodies against HER2 have recently been developed and used in preclinical animal models to evaluate the in vivo expression of the protein. SPECT/micro-CT imaging and ex vivo analyses were used to verify that ^177^Lu-labeled anti-HER2 nanobodies indeed showed high activity in tumors expressing intermediate HER2 levels [55]. In a tumor model expressing high HER2 levels, the construct showed tumor uptake values of 5.99 ± 0.63, 5.12 ± 0.17, 2.83 ± 0.36, and 2.47 ± 0.38% IA/g at 1, 3, 24, and 48 h post-injection, respectively. For this particular study, the ^177^Lu-labeled nanobody was developed as an alternative radioimmunotherapy treatment of HER2-positive breast cancers, as some cancers showed signs of resistance towards traditional anti-HER2 monoclonal antibodies such as Trastuzumab. The study’s measurements yielded an impressive tumor-to-background ratio and did not exhibit specific binding in the HER2-negative model, indicating the nanobody’s potential as both a radioimmunotherapy and imaging agent for HER2-positive breast cancers [55].

**Figure 4:**
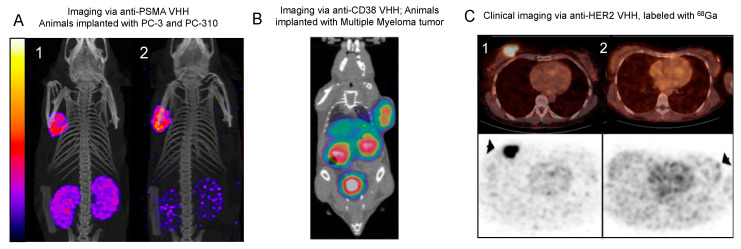
Imaging cancer markers using nanobodies. (**A**) SPECT/CT images of mice bearing PC-3 (right shoulder; PSMA negative) and PC-310 (PSMA expressing) (left shoulder) tumors. Images were acquired 3 h (1) and 24 h (2) after injection of the ^111^In-labeled anti-PSMA nanobody with co-injection of gelofusine and lysine. No uptake was observed in the PC-3 tumor on the right shoulder, while PSMA-expressing PC-310 could be visualized [59]. (**B**) Immuno-PET imaging of subcutaneous multiple myeloma. The probe rapidly accumulated within the tumor; images acquired 1 h post-injection [60]. (**C**) HER2 imaging using nanobody was translated in a Phase I clinical trial [58]. Uptake of ^68^Ga-HER2-nanobody for assessment of HER2 expression in breast carcinoma by PET/CT (top) and PET (bottom). Patient 1 showed the highest tracer uptake (SUV mean 11.8) while patient 2 showed no uptake (SUV mean 0.9). Both patients were scored 3+ after HER2 IHC analyses.

In another study, an anti-HER2 nanobody was radiolabeled with N-succinimidyl-4-[^18^F] fluorobenzoate ([^18^F]-FB) and used to improve PET imaging of tumor-bearing animals [56]. In vivo studies of mouse and rat models yielded high-contrast PET images, with the [^18^F]-FB-anti-HER2 nanobody mediating a high specific uptake in HER2-positive tumors. The short, ~110 min biological half-life of ^18^F is ideal for labeling nanobodies [57], and can generate high signal-to-noise PET images following only ~1–3 h post administration [56]. The potential of the [^18^F]-FB-anti-HER2 nanobody to be translated into the clinic is supported by how its simultaneous administration with Trastuzumab does not compromise its accuracy in capturing tumor cell HER2 expression, as the two antibodies target non-overlapping epitopes [56]. Similarly, an anti-HER2 nanobody radiolabeled with ^68^Ga yielded encouraging results in a Phase I clinical trial involving 20 women with primary or metastatic breast carcinoma [58]. In the majority of pre-identified metastatic disease sites, tracer accumulation surpassed the background level, although primary lesions were often less predictable (Figure 4). With regard to the biodistribution of the ^68^Ga-NOTA-anti-HER2 nanobody, a relatively high uptake was observed in the kidneys, liver, and intestines, but a low background level was noted in all other organs typically affected by breast cancer metastases [58]. This high tracer accumulation specifically in HER2-positive metastases and not in the surrounding native tissues is a reassuring sign that encouraged the trial’s transition into a Phase II study. The evaluation of this ^68^Ga-anti-HER2 nanobody is ongoing in Phase II clinical trials, with the objective of studying uptake in brain metastases of breast carcinoma patients (NCT03331601).

#### 4.1.3. HER3

The human epidermal growth factor 3 (HER3), part of the same family as HER2, is another attractive target for the development of new immunotherapies. The entire family of receptors is known to have a notable effect on tumor progression, and importantly, patients who often develop resistance to traditional cancer treatments also demonstrate higher activated levels of HER3 [61]. Though HER3 is expressed at lower levels in tumors than HER2, targeting and blocking it could still provide valuable help in overcoming resistance and ultimately controlling tumor progression. Although monoclonal antibodies have previously been developed for this purpose, many demonstrate poor tumor penetration, long half-lives, and consequentially low specificity towards HER3.

To overcome some of these concerns and harness the therapeutic potential of nanobodies, a bi-paratopic nanobody construct (MSB0010853) was developed to target HER3, with two domains directed against this receptor and a third designed to bind to albumin, effectively extending its serum half-life [62]. The primary objective of developing of this complex is therapeutic, but further adapting it for noninvasive imaging purposes would provide more insight into its patterns of biodistribution and tumor uptake. To investigate, the nanobody complex was radiolabeled with ^89^Zr and injected in BALB/c mice bearing either FaDu human H441 lung cancer (high HER3 expression) or Calu-1 (no HER3 expression) tumor xenografts. Uptake of the ^89^Zr-MSB0010853 construct was found to be directly correlated with HER3 expression and almost three times higher in H441 tumor xenografts than Calu-1 xenografts, thereby confirming its potential for both therapeutic and imaging applications.

#### 4.1.4. CEA

Carcinoembryonic antigen (CEA) is a β-glycoprotein whose expression is upregulated in inflammatory diseases and various carcinomas, primarily those affecting the colon, lung, and thyroid [63,64]. Radiolabeled monoclonal antibodies have been developed for the diagnosis and treatment of these diseases, but their pharmacokinetics—with slow blood clearance rates and high liver uptake—have made them unsuitable for use as imaging agents. To overcome these concerns, an anti-CEA nanobody was developed and loop-grafted onto a humanized nanobody framework [65]. After being radiolabeled with ^99m^Tc, the nanobody’s potential for whole-body SPECT imaging was tested on animals bearing CEA-positive LS174T colon carcinoma cells. Analyses of flow cytometric and ELISA assays confirmed the nanobody’s ability to specifically recognize and bind to CEA-transfected cells and soluble CEA protein, respectively. The agent’s specificity was coupled with rapid renal clearance, and pinhole SPECT/micro-CT experiments suggested low background levels in every organ except the kidneys [65]. Thus, this ^99m^Tc-labeled nanobody demonstrates a promising possibility of resolving the problematic biodistribution and pharmacokinetics associated with using radiolabeled mAbs for cancer imaging, and therefore shows potential to be translated into the clinic.

#### 4.1.5. PSMA

Glutamate carboxypeptidase II, or prostate-specific membrane antigen (PSMA), is an enzyme that is overexpressed in prostate cancer and a potential target for therapy and therefore molecular imaging [66]. A novel anti-PSMA nanobody was developed via the immunization of a llama with the human PCa cell line, which was then followed by radiolabeling with ^111^In for SPECT/CT imaging [59]. The specificity in tumor binding was evaluated in vivo using mice simultaneously bearing PSMA-negative PC-3 tumors and PSMA-positive PC-310 tumors. The nanobody construct demonstrated specific binding to the PSMA-expressing patient-derived xenografts (PDXs), but not to the PSMA-negative PDXs (Figure 4). As expected, rapid blood clearance was observed for the radiolabeled nanobody. The tracer uptake within the PC-310 tumor provided clear visualization, with a low renal uptake of <4% injected dose per gram. Altogether, these results suggest promising features—fast blood clearance and minimal nonspecific binding—that advocate for the translation of the anti-PSMA nanobodies to clinical tumor imaging applications.

#### 4.1.6. HGF

C-Met and its associated ligand—hepatocyte growth factor (HGF)—are often found in cancer patients with increased tumor aggressiveness and, consequently, poor prognoses. Imaging and characterizing the expression of HGF would therefore provide valuable information in managing the treatment of affected patients. Two anti-HGF nanobodies have thus been developed for both therapeutic and imaging purposes [67]. These nanobodies were fused with an albumin-binding nanobody unit to increase their biological half-life, and then radiolabeled with ^89^Zr. Following radiolabeling, the resulting construct was injected into mice bearing glioblastoma (U87 MG) xenografts. The nanobodies showed selective tumor targeting with similar biodistribution patterns. Uptake in tumor tissue was thus higher than in all surrounding benign tissues except for the kidneys, as the primary clearance organ. This study concludes that these nanobodies have the potential to bring a positive impact both in therapeutic and imaging settings. Future studies analyzing the ^89^Zr-anti-HGF nanobody’s ability to discriminate between tumors with high and low HGF expression could lead to better informed clinical decisions related to patient treatment pathways.

#### 4.1.7. CD20

Immunotherapies targeting the B-lymphocyte antigen CD20 (gene name: MS4A1, or Membrane Spanning 4-Domains A1) are currently the standard care for refractory or relapsing CD20-positive lymphomas [68]. However, a limitation of this treatment is the inability of the full-size antibody agents to thoroughly and effectively penetrate tumors. Nanobodies may circumvent this limitation [69]. Two anti-CD20 nanobodies were radiolabeled with ^68^Ga and indeed demonstrated specific tumor targeting with low background signals in comparison to those of full-size antibodies [70]. Results from this study confirmed the potential of nanobodies as therapeutic and imaging agents. Detecting the presence of CD20 in non-Hodgkin’s lymphomas would further provide valuable information in the detection of low-grade small lymphocytic and marginal zone lymphomas. This is necessary because the [^18^F]FDG PET tracers that are normally used for this purpose have sensitivities of less than 50% in lymphomas [71]. The radiolabeled-nanobody construct may also be useful in monitoring treatment response, including in B-cell-mediated autoimmune diseases, where hCD20-positive B cells are an essential actor of the pathogenesis.

#### 4.1.8. CD38

CD38 (gene name: CD38 molecule) is a glycoprotein of the B cell lineage that is overexpressed specifically in many cases of multiple myeloma (MM) [72], a blood-borne malignancy that remains incurable to this day. Current diagnostic methods are carried out in an invasive manner—from bone marrow aspiration to obtaining biopsies for flow cytometric analyses. Not only would noninvasive alternatives be more convenient for patients, but they would also provide a more holistic, less local landscape of disease progression. This could help stratify specific cases that would benefit the most from therapy, as predicting patient responses to treatment has also been relatively unreliable [60]. CD38, identified within positive MM flow cytometry examinations, has recently been studied as a target of the diseased cells, aiding in the diagnosis and treatment monitoring of the condition. The development of novel, sensitive techniques to monitor and recognize CD38 expression in a noninvasive manner could provide great insight into MM progression as well as the efficacy of the anti-CD38 treatment response.

Monoclonal antibody-based immunomodulatory therapeutics have been developed to specifically target CD38, the most notable being daratumumab [73]. Daratumumab has seen significant success in the past few years and has even been radiolabeled to extend its use to imaging preclinical MM models via immuno-PET. However, the size of the mAb (~160 kDa) is larger than the typical cutoff size seen for glomerular filtration (~60 kDa) [60]. This meant that daratumumab had to be labeled using radionuclides with long half-life characteristics, such as ^89^Zr and ^64^Cu, in order to complement the lengthy circulation time of the mAb. Binding instability in radionuclide–mAb complexes can lead to unbound radioisotopes, which ultimately reduces uptake specificity to MM cells within the bloodstream [60].

To overcome these challenges, an anti-CD38 nanobody was radiolabeled with ^68^Ga as an alternative for the molecular imaging of MM [60]. The merit of this nanobody was tested in mice bearing either subcutaneous or disseminated (orthotopic) MM.1S xenografts. The ^68^Ga-anti-CD38 nanobody demonstrated rapid accumulation in tumors (1.76 ± 0.305%ID/g (*n* = 5)) 1 h post-injection, which was accompanied by a promising tumor-to-bone ratio (TBR = 5.79) as well as evidence of rapid renal clearance (Figure 4). Comparatively, the uptake of [^18^F]FDG, currently the gold standard radiotracer for PET imaging of MM [74], was highly nonspecific with a far lower TBR of 0.39 [60]. This general uptake in the bone explains why nonspecific probes require additional invasive biopsies of the bone marrow to fully confirm diagnosis. Immuno-PET imaging of disseminated MMs using the ^68^Ga-anti-CD38 nanobody delineated bone lesions as early as 3–4 weeks after tumor cell inoculation in mice. Preloading with daratumumab, interestingly, led to a significantly reduced uptake of the nanobody in disseminated bone lesions, suggesting that they may bind to overlapping epitopes. This is an important factor to consider as these ^68^Ga-anti-CD38 nanobody-based imaging probes could potentially also help with predicting patient response, reliably identifying those most suitable for daratumumab treatment.

Overall, the ^68^Ga-anti-CD38 nanobody-based probe was able to recognize all subcutaneous and orthotopic MM lesions to a better extent than the control probes, demonstrating high radiochemical yield (>50%), purity (>99%), and immunoreactivity (>95%) [60]. This tool for molecular imaging of MM has been found to be applicable in other lymphomas expressing CD38 and holds potential to be used for stratification of solid tumors [60]. Therefore, CD38-nanobody imaging is a strong candidate for translation into the clinic because of its potential to help diagnose MM at its earlier stages, assess treatment response, detect MM-affected bones without causing bone destruction, and allow for same-day imaging with a higher TBR than daratumumab or other mAb-based imaging probes.

#### 4.1.9. Mesothelin

Mesothelin (gene name: MSLN) is a cell surface glycoprotein that is typically present on mesothelial cells, such as those that line the pleura, peritoneum, and pericardium [75]. It is a tumor differentiation antigen that has been found to be overexpressed in many cancer types, including mesothelioma, lung adenocarcinoma, and triple-negative breast cancer, the latter of which is difficult to treat due to its resistance to hormone-based therapies and Trastuzumab [75,76]. Therapies targeting mesothelin are currently being developed but identifying the subsets of patients eligible for these therapies has long remained a major challenge. Two nanobodies (A1 and C6) radiolabeled with ^99m^Tc were developed as non-invasive imaging agents and evaluated in the mesothelin-positive HCC70 breast cancer cell line against a mesothelin-negative MDA-MB-231 cell line [76]. The two nanobodies showed specific, high affinity binding to mesothelin in the in vitro studies that were conducted [76]. In SPECT images generated from xenografted models, the signal from MDA-MB-231 was 5-fold lower than in HCC70 tumors for the ^99m^Tc-A1 construct, and 1.5 times lower for the ^99m^Tc-C6 construct. Both resulted in high tumor-to-background ratios, but the ^99m^Tc-A1 nanobody in comparison proved to be the more promising tool for translation into the clinic due to its higher affinity and higher absolute tumor uptake (up to 1.5-fold in vivo) [76].

#### 4.1.10. ECM Biomarkers: Imaging Cancer

The extracellular matrix (ECM) plays an important role in nearly all tissues of the body, as it helps provide scaffolding for internal cells and is involved in the biochemical signaling pathways that regulate core cellular functions, such as cell–cell adhesion, differentiation, and proliferation [77,78]. As such, the ECM is also a central component of the tumor microenvironment (TME), where it interacts with nearby tumor cells to aid in cancer survival through angiogenesis, invasion, and developed resistance against different therapeutics [79,80]. Targeting ECM proteins for imaging or therapeutic purposes is an attractive option: they have an inherent stability against mutations, which protects against common immuno-PET imaging difficulties associated with tumor cell heterogeneity, genomic instability, and immunoediting.

A nanobody was developed to specifically target an alternatively spliced domain of fibronectin (FN-EIIIB) expressed in the ECM and neovasculature of many diseases, including cancer [80]. An immediate benefit of using EIIIB in the preclinical setting is the fact that its sequence is identical in both mice and humans, which means murine models will likely be very representative of human responses. To visualize and monitor activity via immuno-PET/CT imaging, the FN-EIIIB-specific nanobody was labeled with ^64^Cu that allowed imaging of the nanobody’s dynamics in vivo. When this nanobody was injected into mice with triple-negative breast cancer (TNBC), in vivo PET/CT signals analyzed 2 h post injection demonstrated highly specific binding to the ECM of primary tumors and lung metastases [80]. No nonspecific uptake was seen in the livers or lungs of control mice. This same anti-ECM nanobody showed similarly promising results in mice with melanoma and pancreatic ductal adenocarcinoma (PDAC), the latter of which has historically been difficult to detect in its early stages [81]. Following ^64^Cu-FN-EIIIB nanobody injection into mice with PDAC, immunohistochemical analysis demonstrated EIIIB expression not only in the stroma of PDAC tumors, but also in early pancreatic intraepithelial neoplasia (PanIN) lesions [80]. This signal was absent in the pancreas of healthy mice. Uptake of the ^64^Cu-anti-EIIIB nanobody was 18-fold higher for PDAC and 7-fold higher for PanIN compared with that of the control mice. PET/CT images generated using this nanobody as an imaging probe had higher resolution, clarity, and signal-to-noise ratios than the images obtained using traditional [^18^F]FDG PET imaging.

Taken together, imaging cancer-specific markers is important in analyzing expression patterns in vivo, predicting the trajectory of diseases, as well as better informing treatment decisions. Since markers such as those described above are specifically overexpressed on cancer cells and not highly expressed on native cells, nanobody targeting demonstrates high specificity, ultimately allowing for the generation of high-resolution images with promising signal-to-noise ratios. Radiolabeling nanobodies in place of antibodies or larger imaging moieties has proven to be reliable due to faster blood clearance, quicker image acquisition, higher binding affinity, and specific tumor uptake, demonstrating their potential to be translated into the clinic and used as potent cancer imaging agents. Of the markers discussed in this section, especially the HER2, CD38, and ECM markers are discussed in greater detail, as the number of available studies concerning these is more substantial than for other cancer cell markers (Table 1).

### 4.2. Imaging Immune Checkpoint Markers

#### 4.2.1. Background

Immune checkpoint blockade immunotherapy has revolutionized the treatment of a spectrum of malignancies, such as advanced melanoma and non-small cell lung cancer, among others [82,83,84]. Its premise works to reinvigorate exhausted T cells and other anti-tumor immune cells through helping them activate, expand, and continue to proliferate and penetrate tumor areas. However, responses to immunotherapeutic treatment remain mixed and unpredictable among patients. Moreover, some patients face serious side effects related to excessive activation of the immune system, some of which, such as myocarditis or pneumonitis, can be life-threatening [85,86,87]. Therefore, evaluating ongoing responses to treatment and identifying cohorts of patients that may or may not respond to a particular treatment remains a critical challenge. Another common consequence of checkpoint blockade immunotherapy is pseudoprogression, which is the false impression of tumor growth resulting from sudden and elevated immune cell infiltration and expansion. Pseudoprogression is often misleading because it is indistinguishable from cancer progression when seen through the perspective of anatomical MRI or CT images. Developing methods to address this issue is therefore of tremendous value. To tackle this, noninvasive imaging of the TME using radiolabeled antibodies and antibody fragments against specific immunological markers have been developed and used extensively in both preclinical and clinical settings. Such molecular imaging methods have the potential to improve immunotherapeutic monitoring by providing insight into the biological processes in the TME both before initiation and during treatment, which can be used to identify patterns of responses and even predict treatment response.

#### 4.2.2. PD-L1

Programmed death-ligand 1, or PD-L1 (gene name: CD274), is a cell-surface protein typically expressed in normal tissues to balance and downregulate immune response and recognition by cytotoxic T lymphocytes (CTLs). This process is mediated by the interaction between PD-L1 and programmed cell death protein 1 (PD-1), an immunoregulatory protein found on the surface of CTLs that, when bound by PD-L1, suppresses its cytotoxic activity [88]. This interaction is hindered by checkpoint blockade therapies, therefore triggering T cell (re)activation that can lead to the killing of cancer cells.

Expression of PD-L1 on cancer cells has been shown to be correlated with treatment outcome [89]. Although they are only effective in 20–40% of patients, many cancer prognoses have been seen unprecedented improvement through the inhibition of the PD-1/PD-L1 axis [90]. However, PD-L1 expression is often heterogeneous, even within the same tumor, which makes imaging its entire cancer cell expression landscape very valuable [91].

PD-L1-targeting antibody fragments have recently been developed and used to generate high-contrast images of tumors with varying levels of PD-L1 expression [92,93,94]. Four high-affinity anti-PD-L1 nanobodies were radiolabeled with ^99m^Tc, and evaluated for preclinical SPECT/CT imaging of PD-L1 in syngeneic mice [92]. Two of the four tested nanobodies showed specific antigen binding and successfully differentiated between PD-L1-positive and PD-L1-negative lung cancer cells. The intensity of the observed signal was concentration dependent, as high PD-L1 levels generally correlated with stronger signals.

Another study developed an anti-mouse PD-L1 nanobody and used both ^18^F-labeled and ^64^Cu-labeled VHH to image PD-L1-expressing cells in vivo [95]. The radiolabeled VHH was injected and used for whole-body PET/CT imaging of B16 melanoma-bearing animals. The radiolabeled nanobody detected the tumors with great clarity. Of note, the authors discovered that brown adipose tissue (BAT) expresses high levels of PD-L1, suggesting PD-L1’s potential role in metabolic function, now a topic of ongoing research [96,97]. This discovery highlights the value of whole-body PET imaging techniques to reveal all tissues expressing the target molecule beyond what is already known or expected, thereby leading to further advancements in scientific knowledge.

Another study used a nanobody-based probe to assess human PD-L1 expression via PET imaging, and then investigated the consequences of non-specific chelator installation on nanobodies when the VHH has lysine residues within the complementarity-determining regions (CDRs) of the nanobody [94]. The construct was conjugated with the NOTA chelator either randomly on its lysine residues or site-specifically using the sortase A enzyme. Following preparation, ^68^Ga-NOTA-(hPD-L1) nanobodies were used for the relevant in vivo analyses. Results collected ~90 min after injection showed specific tumor uptakes of 1.77 ± 0.29% IA/g for the randomly conjugated VHH and 1.89 ± 0.40% IA/g for the site-specifically conjugated VHH. The notable stability of both conjugates demonstrates that random lysine conjugation could potentially be an attractive strategy encouraging clinical translation of the radiolabeled nanobody.

**Figure 5:**
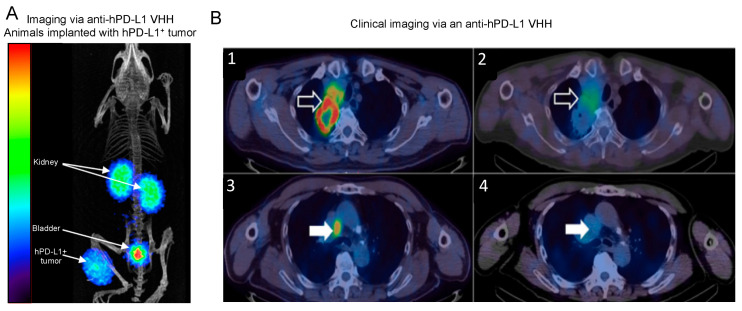
Imaging of checkpoint markers. (**A**) Imaging of human PD-L1. PET/CT imaging ~90 min p.i. of the ^68^G-labeled anti-hPD-L1 VHH of a mouse bearing the hPD-L1^+^ tumor [94]. (**B**) Phase I study of ^99m^Tc-labeled anti-hPD-L1 VHH in non-small cell lung cancer patients [98]. The right upper lobe tumor (**1**) shows high [^18^F]FDG uptake and (**2**) ^99m^Tc-VHH uptake within the tumor. A mediastinal lymph node showed high [^18^F]FDG uptake (**3**), however low ^99m^Tc-VHH uptake was observed (**4**), suggesting PD-L1 is heterogeneously expressed between the primary tumor site and distant sites of disease within the same patient.

There is an ongoing clinical trial (NCT02978196) aiming to evaluate the safety, dosimetry, and efficacy of an ^99m^Tc-labeled anti-PD-L1 nanobody in non-small cell lung cancer (NSCLC) (Figure 5) [98]. These factors are being compared to tissue biopsies, the gold standard method for evaluating and quantifying expression of PD-L1 in tumors. Another clinical trial (NCT03638804) is currently using an ^89^Zr–Fc fusion nanobody (Envafolimab) to analyze targeted uptake and biodistribution in human subjects carrying PD-L1-positive tumors [99,100]. This monitoring is being done via PET imaging, and factors such as the safety and necessary dosimetry of this Fc fusion nanobody are also being evaluated.

Of note, radiolabeled full-sized anti-PD-L1 antibodies have been used in the clinic as well. One study used ^89^Zr–atezolizumab on 22 patients of different tumor types, including metastatic bladder cancer, non-small cell lung cancer, and triple-negative breast cancer [101]. The PET signal was detected at primary lesions and all main metastatic sites, especially from bladder cancer patients. Strikingly, the PET signal uptake was highly correlated to patient response to treatment, as measured by RECIST categorization and Kaplan–Meier curves, and at a more significant degree compared to two separate FDA-approved tumor tissue PD-L1 immunohistochemistry methods, suggesting the potential of molecular imaging for the assessment of PD-L1 expression and treatment prognoses.

Overall, there is significant promise associated with imaging PD-L1, and the unique properties of nanobodies make them an attractive choice to address this clinical need.

#### 4.2.3. CTLA-4

Cytotoxic T-lymphocyte antigen-4 (CTLA-4) is a checkpoint inhibitory molecule expressed on the cell membrane of activated T cells. Similar to PD-1/PD-L1, CTLA-4 molecules have a critical immunoregulatory role through their ability to suppress T cell cytotoxicity but differ in that they do so by interacting with the B7 family costimulatory molecules, CD80 and CD86, on the surface of antigen-presenting cells (APCs). Ipilimumab, the first anti-CTLA-4 antibody that was used and later approved in the clinic, has long demonstrated great benefit in patients with locally advanced and metastatic melanoma [102]. Since the approval of Ipilimumab in the clinic, it has been used in combination with PD-1/PD-L1 blockade as a powerful way to treat a spectrum of different malignancies, including non-small cell lung carcinoma [103]. Analyzing the expression level of CTLA-4 in the TME and delineating whether it has correlation with the treatment outcome is thus of great importance. However, measuring the CTLA-4 levels using patient biopsies has revealed high variability even between different areas of the same tumor, as well as between primary and secondary lesions [104]. Noninvasive imaging has great potential to address this issue. A recently developed method uses a CTLA-4-specific nanobody-fluorescent carbon quantum dots complex (QDs-Nb) in order to detect CTLA-4-positive cells by assays such as flow cytometry and immunofluorescent staining [105]. This complex proved to be sensitive, as the number of CTLA-4-positive cells detected using the QDs-Nb probe in both tumoral tissue and adjacent mucosa was significantly higher than that detected when using the anti-CTLA-4 mAb. Furthermore, this approach showed no significant toxicity in vitro or in vivo, suggesting potential for application to detect targets with low expression.

CTLA-4 expression has also been assessed through PET imaging using an anti-CTLA-4 nanobody. The nanobody was PEGylated to improve the signal-to-noise ratio and decrease kidney uptake [106]. The radiolabeled-PEGylated nanobody was injected into mice bearing B16 melanoma, and the images delineated the radiolabeled nanobody’s detection of the tumors. The TMEs of these mice had circulating anti-CTLA-4 mAbs (clone 9H10) that had been administered for therapeutic purposes, but the CTLA-4 molecules were still detectable using the nanobody. Furthermore, there was no significant difference in the overall survival between mice imaged with ^89^Zr-labeled nanobodies and control animals that were not imaged, suggesting that the imaging would not interfere with the efficacy of the anti-CTLA-4 treatment.

#### 4.2.4. LAG-3

In addition to CTLA-4 and the PD-1/PD-L1 axis, other immune-modulating molecules have been extensively explored for their potential therapeutic value. Of particular interest has been the lymphocyte-activation gene 3 (LAG-3). LAG-3 is a protein expressed on tumor-infiltrating lymphocytes (TILs). Similar to PD-1 and CTLA-4 checkpoint molecules, its function is to provide signals that downregulate T cell activity and replication, through a mechanism of interacting with the major histocompatibility complex II (MHC-II) molecules of APCs [107]. Therefore, an increased level of LAG-3 expression is often associated with T cell exhaustion, leading to lower T cell activity and decreasing their ability to activate or secrete anti-tumor cytokines [108]. High expression of the LAG-3 marker in patients is typically correlated with shorter progression-free survival upon treatment with checkpoint blockade therapy [109]. Therefore, imaging expression of LAG-3 in the TME has potential diagnostic and prognostic value.

LAG-3-specific nanobodies were developed and radiolabeled to assess LAG-3 expression in tumor-bearing animals [110]. Imaging demonstrated highly specific uptake in immune peripheral organs, with no specific signal detected in the LAG-3 gene knockout mice [110]. Moreover, the uptake correlated with the presence of LAG-3 following assessment with immunohistochemistry and flow cytometry. To further confirm the utility of this anti-LAG-3 nanobody in clinical care, experiments were performed by subcutaneously implanting LAG-3-positive tumor cells into immunocompromised mice. Results showed that all tested nanobodies were indeed able to distinguish tumors expressing LAG-3. A follow-up study in tumor-bearing animals receiving PD-1 blockade treatment found that nanobody-LAG-3 imaging can have prognostic value in predicting the response to checkpoint blockade [111]. The result was encouraging as it suggests that the nanobody could be used in the clinic to assess LAG-3 expression both before and during therapy.

Altogether, these studies propose the possibility that radiolabeled nanobody-based probes could be used as effective imaging agents to target checkpoint molecules, improving upon the shortcomings of checkpoint blockade immunotherapies currently used in treating cancers. Attempting to get homogeneous, predictable responses to these checkpoint blockade treatments has been a large obstacle for many years. This issue is especially important to overcome because the expression of many checkpoint molecules has been found to be correlated with treatment outcome. Ultimately, such imaging experiments will help physicians make more well-informed and personalized decisions for patients.

### 4.3. Imaging Immune Markers

#### 4.3.1. Background

The response to immunotherapy is directly representative of changes within the tumor immune landscape. Gaining better insight into the dynamics of treatment can therefore shed light onto the widely heterogeneous response to immunotherapeutics. Furthermore, monitoring immune responses can be used as a proxy to assess an ongoing response to treatment, and may provide valuable insight to predict patient responses. Therefore, noninvasive imaging approaches to image specific subsets of immune cells, such as macrophages and T cells, have been developed and recently translated into the clinic [112,113]. Nanobodies are an ideal tool to address some of these issues, and thus several VHHs targeting immune markers have been developed and used to image immune responses.

#### 4.3.2. CD8

CD8+ T cells play a central and indispensable role in anti-tumor immune responses. Checkpoint inhibitors have written a new chapter in patient management by (re)activating CD8+ T cells and enabling them to kill cancer cells [114]. A better understanding of the dynamics of CD8+ T cells (location, expansion, and number), gaining further insight into the mechanisms behind their activation, and their ability to infiltrate the TME may help to better comprehend the heterogeneous response to treatments. Imaging CD8 T cells can help address some of these issues.

**Figure 6:**
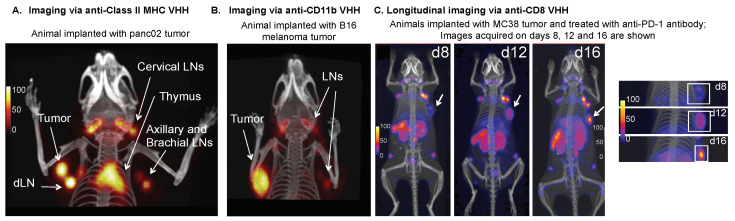
PET-CT scans of tumor-bearing animals imaged with radiolabeled nanobodies. (**A**) ^18^F-labeled anti-class II MHC nanobody detects very small tumors and lymphoid organs with great clarity [117]. (**B**) ^18^F-labeled anti-CD11b nanobody detects B16 melanoma tumors by virtue of detecting tumor-infiltrating myeloid cells [118]. (**C**) ^89^Zr-labeled PEGylated anti-CD8 nanobody can be used to longitudinally monitor the dynamics of CD8^+^ cells in response to PD-1 blockade [115]. White boxes show the tumor. For more images and data-quantification, see [43,115,118].

To noninvasively image the dynamics of CD8+ T cells, an anti-CD8 (CD8α) nanobody was developed [43]. To analyze the in vivo characteristics of the nanobody, it was first labeled with a fluorophore (Alexa647) and then injected into mice. Lymphoid organs were excised two hours post-injection with flow cytometric analysis confirming specific binding of the nanobody to CD8+ T cells with high specificity and affinity. The nanobody was then radiolabeled with ^89^Zr and used to noninvasively image the animals, with PET revealing the lymphoid organs with clarity [43]. A PEG moiety was added to the radiolabeled nanobody, improving the signal-to-noise and decreasing kidney uptake. To explore how the dynamics of the CD8+ T cells changed in response to checkpoint blockade therapy, animals bearing B16 melanoma were treated with CTLA-4 blockade and then subjected to CD8-PET imaging longitudinally over the course of four treatments on days 9, 16, 23, and 30 following tumor inoculation. Response to CTLA-4 blockade was correlated with a homogenous distribution of the CD8-PET signal, whereas non responders displayed a more heterogeneous infiltration of CD8+ T cells. These results taken from the melanoma model were further confirmed by two different breast cancer models as well as an MC38 colorectal cancer model that had been responsive to PD-1 blockade (Figure 6) [115]. Together, these results suggest that successful checkpoint blockade therapy is often accompanied with the expansion and infiltration of CD8^+^ T cells into the tumor core. This is consistent with what has previously been observed in clinical studies [116].

More recently, anti-human CD8 nanobodies have been developed and characterized in vivo in preclinical mouse models [119]. MC38 tumors were engineered to express human CD8, and the radiolabeled anti-human CD8 nanobodies was used to image the hCD8^+^ tumors. Results showed the radiolabeled nanobody was able to detect the presence of CD8+ cells in vivo, encouraging wider translation of the approach into clinical settings [120]. Of note, a minibody against CD8 has now been translated into the clinic, where the Phase I study has been completed and Phase II is currently being planned [113]. Overall, these studies suggest the potential of CD8 immuno-PET imaging for the assessment of immunotherapeutic efficacy, which may ultimately help in predicting patient responses.

#### 4.3.3. MHC Class II

Tumors are infiltrated with immune cells such as macrophages and T cells. Therefore, tracking the infiltration of immune cells is an attractive approach to detect tumors. A nanobody against mouse class II major histocompatibility complex (MHC) was developed and used to noninvasively image lymphoid organs and detect tumors. The ^18^F-labeled nanobody was cleared from circulation rapidly and images, acquired 2h post-injection of the radiolabeled VHH, revealed lymphoid organs with great clarity. Animals implanted with syngeneic or xenogeneic tumors were subjected to class II MHC-PET imaging, where images identified tumors through detecting tumor-infiltrating class II MHC+ cells with great clarity [117,118]. Of note, this construct was more sensitive in detecting small tumors than [^18^F]FDG (Figure 6), which is often considered the gold standard, suggesting that this approach can be a complementary method to the clinically used [^18^F]FDG PET imaging [117].

To non-invasively image the human immune response in graft versus host disease (GvHD), a nanobody specific for human class-II MHC was developed [121]. Noninvasive imaging of the human immune system was conducted using bone marrow–liver–thymus (BLT) mice that develop graft-versus-host disease (GvHD), mediated by infiltration of activated human T cells into major organs such as the liver. GvHD is a major, often lethal complication of allogeneic hematopoietic cell transplantations where the donor’s T cells (which make up the “graft”) are incompatible with the patient’s native cells (the “host”) [122]. This causes them to initiate an immune response against the patient cells, treating them as if they were foreign. GvHD often limits treatment options and there currently is no reliable way of preventing or taming it.

To potentially diagnose GvHD, the BLT mice in the study were imaged with an anti-MHC-II ^64^Cu-labeled nanobody to detect the activated and infiltrating T cells responsible for the GvHD development [121]. BLT mice with Stage 3 GvHD were then imaged by PET/CT, and an intense signal was found in the liver. This contrasted the fact that the signal was imperceptible in mice without GvHD as well as in the control NOD/SCID mice. All organs were dispersed into cell suspension to detect the source of the MHC-II signal by flow cytometry, and an increased human T cell infiltration was subsequently observed in the livers of GvHD-positive BLT mice. These results demonstrate the potential of noninvasive imaging and nanobodies to better inform diagnoses and evaluate the immune system’s role in GvHD and other diseases characterized by inflammation.

#### 4.3.4. MMR

The many immune cells involved in the TME are key elements in cancer progression and resolution, due to their dynamic ability to induce tumor progression from an initially supportive or hostile state. During the earliest stages of tumor development, myeloid cells are attracted to the tumor stroma. These infiltrating immune cells can play different—at times, even opposite—roles. For example, MHC-II^hi^ Tumor Associated Macrophages (TAMs), referred to as M1-like macrophages, can help shape the TME into an anti-tumor status, whereas MHC-II^low^ TAMs, referred to as M2-like macrophages, facilitate angiogenesis and are categorized as pro-tumoral. The macrophage mannose receptor (MMR; gene name: CD206) has been found to be overexpressed in these M2-like tumor-promoting TAMs [123]. To monitor the dynamics of MMR+ TAMs in tumors, an anti-MMR nanobody, cross-reactive with mouse and human CD206, was developed and radiolabeled with ^18^F and then injected into 3LL-R tumor-bearing mice [123]. Negligible retention was observed in MMR-deficient hosts whereas regions expressing MMR, including tumors, showed specific uptake of the radiotracer (2.40 ± 0.46% IA/g). The tumor uptake was also significantly lower in CCR2-deficient mice than in wild-type mice, confirming the correlation between the number of TAMs and the extent of the nanobody uptake. This also shows the tracer’s ability to specifically target tumor-infiltrating macrophages. Furthermore, ex vivo autoradiography showed that the tracer could detect and image the heterogeneity of the MMR expression throughout the TME, as its biodistribution patterns were heterogeneous at the tumor borders as well as in intra-tumoral hotspots [123]. Because of the correlation between the presence of MMR-expressing TAMs and tumoral progression, the ^18^F-nanobody could hold clinical value as a prognostic tool. These studies indicate that imaging of MMR^+^ macrophages in the TME is feasible, and future studies will investigate whether it has predictive value for cancer immunotherapy. Of note, the anti-CD206 nanobody has moved into clinical trial (NCT04168528) [124].

#### 4.3.5. CD11b

The TME consists of a complex mixture of immune cells. CD11b (gene name: ITGAM, Integrin Subunit Alpha M) is a particularly relevant marker because it is largely expressed on the surface of myeloid cells, such as macrophages, monocytes, neutrophils, granulocytes, and natural killer cells [125]. Imaging CD11b molecules would thus give a global snapshot of the myeloid immune response in the TME and can be used as a general marker to image inflammation in disease.

An anti-CD11b nanobody was developed and characterized both in vitro and in vivo [118]. The anti-CD11b nanobody was radiolabeled with the ^18^F radionuclide, and PET imaging of C57BL/6 mice implanted with B16 melanoma cells showed that the construct could detect CD11b^+^ tumor-infiltrating cells (Figure 6). The ability of the construct to image inflammation was assessed using Complete Freund’s Adjuvant (CFA), a solution known to result in severe inflammation and influx of neutrophils at the site of injection. Following 24 h post injection, the animals were imaged with the ^18^F-nanobody and showed strong PET signals that were localized in the inflamed regions, confirming the nanobody’s ability to track these global immune responses with excellent specificity.

### 4.4. Summary of Imaging Non-Cancer Targets

Many of the most effective cancer treatments are currently based on modulating the immune system, so truly understanding the system’s function and dynamics are essential both with regard to developing new treatments as well as predicting or monitoring patient responses. Imaging modalities, especially those utilizing precise and high affinity probes such as nanobodies, provide a reliable means to achieve this goal.

## 5. Nanobodies as a Tool for Imaging Non-Malignant Disease

In addition to being used as imaging agents for cancer, nanobodies have also been developed against biomarkers that are overexpressed in other diseases, such as fibrosis, atherosclerosis, arthritis, and some neurodegenerative disorders. Many of these diseases currently do not have ways of being effectively imaged or detected, especially in their early or reversible stages. Identifying reliably targetable markers has long been a challenge, along with achieving selective and specific uptake in diseased tissues. There are great benefits associated with overcoming these challenges, as many of these diseases see large, time-sensitive increases in morbidity if left undiagnosed. Noninvasive imaging techniques, especially those mediated by nanobodies, have demonstrated promising potential to image non-cancer markers with high specificity, clarity, and acceptable signal-to-noise ratios, and thus can be used to detect a variety of complications before they spread or become life-threatening, allowing for treatments to be initiated sooner.

### 5.1. ECM Biomarkers: Imaging Fibroses

Nanobody targeted imaging of the extracellular matrix (ECM), specifically using anti-EIIIB VHH, has been discussed above. The FN-EIIIB protein, in addition to cancer, is widely expressed in diseases characterized by ECM deposition, such as fibrosis, aneurysms, and atheromas, while being nearly absent from most normal adult human tissues. As such, the aforementioned advantages of targeting ECM for cancer imaging extends its relevancy across a number of conditions, providing broad applicability.

**Figure 7:**
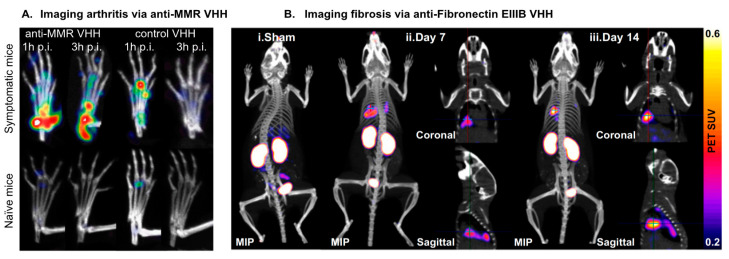
(**A**) Anti-MMR nanobody can be used to detect arthritis in arthritic mice. Images of the affected or healthy limbs were obtained at 1 or 3 h post-injection of ^99m^Tc-anti-MMR VHH or the BCII10 control VHH (see [128]). (**B**) The ^64^Cu-labeled anti fibronectin EIIIB VHH (VHH-NJB2) can detect pulmonary fibrosis in a bleomycin-induced lung fibrosis model with great clarity. The cross-hairs in the sagittal and coronal images show the fibrotic lesions within the lungs. For more details and image quantifications, see [80].

The previously mentioned ^64^Cu-anti-EIIIB nanobody study also investigated the potential of the complex to image and detect pulmonary fibrosis [80]. Fibrotic tissues are also characterized by the increased deposition of FN-expressing ECM, which is distinguishable from normal tissues that are void of FN-containing EIIIB [126,127]. For the study, mice were intratracheally treated with bleomycin, to mimic features of pulmonary fibrosis, and subsequently injected with the ^64^Cu-anti-EIIIB nanobody [80]. PET/CT images showed that the nanobody bound to fibrotic lung regions, with an increased uptake 14 days after bleomycin treatment, comparative to only 7 days post-treatment. In control, sham-treated mice, this increase in uptake was not observed and the PET signal in the lungs was lower than in bleomycin-treated mice. This suggests that the anti-EIIIB nanobody is able to detect pulmonary fibrosis at its early stages in a noninvasive, specific, and clear manner (Figure 7).

The selective accumulation of the anti-ECM nanobody in unhealthy sites of various diseases, especially without background noise or off-target toxicity, shows its potential to be translated into the clinic for imaging and therapeutic applications. This is promising for early detection of time-sensitive diseases, such as pancreatic cancer, in turn improving treatment prognosis and increasing patient survival rates. In addition to their selective abundance in diseased tissues, many ECM-associated proteins and domains can be nanobody-targeted for imaging or therapeutic purposes without inciting an autoimmune response [80]. One of the most significant merits of this anti-ECM nanobody, however, is the applicability of a singular complex across multiple disease types [80].

### 5.2. VCAM-1: Imaging Cardiovascular Complications

Vascular cell adhesion molecule-1 (VCAM-1) is a glycoprotein primarily expressed on endothelial cells that are involved in inflammation-regulated cell adhesion and the transendothelial migration of leukocytes [129]. VCAM-1 performs these functions by binding to α4β1 integrin on the cell surface of leukocytes, which then activates the necessary signaling pathways and induces leukocyte recruitment. Secretion of pro-inflammatory cytokines, such as tumor necrosis factor-α (TNF-α), upregulates the expression of VCAM-1, among other cell adhesion molecules, which has been found to correlate with the progression of immunological diseases and cardiovascular complications [129,130].

An example of such a cardiovascular complication that can be detected from VCAM-1 expression is atherosclerotic disease, which can lead to stroke and myocardial infarction. While there has been some recent progress made in treating the acute or fatal complications of atherosclerosis, it has remained difficult to assess patient risk for the purpose of detecting abnormalities early on [131]. By detecting a clearly atherogenic phenotype before the disease progresses into its later stages, treatment protocols can be better catered towards reversing or stopping plaque formation, which is currently the leading reason for atherosclerotic mortality. Overexpression of VCAM-1 has been proven to precede plaque development, as the recruitment of leukocytes to the vascular wall plays a key part in inducing plaque formation and growth [132,133]. For this reason, full-size antibodies targeting VCAM-1 have been developed in recent years and subjected to contrast-enhanced ultrasound molecular imaging (CEUMI) [134]. This allowed for the close monitoring of VCAM-1 expression in mouse models, which in turn made it clear when atherosclerotic symptoms began to develop. However, these full-size antibodies would not only be costly for human use [135], but the biotin–streptavidin linking of the ligands to their microbubble carriers also raised concerns of possibly binding to endogenous biotin [136]. Therefore, alternative methods involving nanobodies have been tested, and are better candidates for ultimate translation into the clinic.

Anti-VCAM-1 nanobodies were developed and conjugated to microbubbles to detect the VCAM-1 expression levels, both in mouse models of atherosclerosis and ex vivo human endarterectomy specimens [131]. These nanobodies were cross-reactive towards both murine and human VCAM-1, and the purpose of the microbubbles was to be used as a contrast agent for ultrasound imaging. This tracing was done noninvasively using CEUMI, and the results of the anti-VCAM-1 nanobodies (MB_cAbVcam1-5_) were compared to those of the control nanobody (MB_VHH2E7_) [131]. As with the many nanobody imaging agents targeting other markers, the binding specificity of MB_cAbVcam1-5_ to VCAM-1 was also verified using IHC, autoradiography, and in vivo experiments. Not only this, but the anti-VCAM-1 nanobody gave off a strong signal in the aorta of mice with varying stages of atherosclerosis, while yielding minimal nonspecific detection in control mice. MB_VHH2E7_ also did not pick up any significant signals, further confirming the specificity of MB_cAbVcam1-5_. Unlike the previously mentioned full-size antibodies, these microbubble-conjugated anti-VCAM-1 nanobodies are translatable into the clinic and could improve risk stratification for atherosclerosis.

Visualizing VCAM-1 expression was also recently attempted using tracers suitable for noninvasive nuclear imaging [130]. ^99m^Tc-labeled nanobodies were designed to target VCAM-1 and then used to detect aortic arch atherosclerotic lesions in ApoE-deficient mice [130]. It was found after analysis that the ^99m^Tc nanobody had high lesion-to-control (4.95 ± 0.85), lesion-to-heart (8.30 ± 1.11), and lesion-to-blood ratios (4.32 ± 0.48). SPECT/CT imaging techniques also precisely identified where atherosclerotic lesions were located in the aortic arches of the mice [130]. Furthermore, the radiolabeled nanobody’s specificity and uptake in VCAM-1-expressing lesions were confirmed using IHC and a variety of in vivo competition experiments.

These noninvasive methods of imaging VCAM-1 expression using nanobodies shows their potential in improving the prognosis of cardiovascular disease, whether through aiding in early detection or continually assessing risk in already diagnosed patients. Past studies examining the anti-VCAM-1 nanobody’s role as an imaging agent for ultrasound and SPECT/CT scans have demonstrated its usefulness and safety in the clinic, thus making clinical translation both likely and beneficial.

### 5.3. MMR: Imaging Arthritis

Rheumatoid arthritis (RA) is an autoimmune disease that involves the destruction of cartilage and bone by inflammatory cells, particularly in the joints [137]. A prominent issue in treating RA is how difficult it is to detect and monitor its initial stages. Earlier treatment would help minimize structural damage by reversing the inflammatory response more efficiently. Yet, although various imaging techniques have been developed and used for this purpose, they have not been able to provide much information past mere anatomical structures [138]. Visualizing in vivo processes causing damage to the synovial tissues and bones would be more informative with regards to RA pathogenesis but would necessitate novel molecular imaging techniques. To achieve this goal, nanobodies have been developed to target markers expressed in arthritic joints, specifically the macrophage mannose receptor (MMR), as discussed above [123,128,138].

The anti-MMR nanobodies were first radiolabeled with ^99m^Tc and then injected into DBA/1 mice with collagen-induced arthritis (CIA), a representative mouse model of RA [128]. CIA was induced in these mice by injecting a mixture of collagen type II in CFA [128]. SPECT/micro-CT images were then generated in order to test if MMR could be a useful target marker in monitoring and quantifying arthritic progression. Quantitative PCR and flow cytometry confirmed that MMR expression was detected on bone marrow, lymph node, and spleen cells of mice with CIA, as well as on CD11b^+^F4/80^+^ macrophages in the synovial fluid [128]. SPECT images showed the specific uptake of ^99m^Tc-labeled anti-MMR nanobodies in inflamed joints, as control nanobodies targeting irrelevant markers did not show specific uptake (Figure 7). Collectively, these results demonstrate the effectiveness of targeting MMR with nanobodies in order to specifically and reliably image the inflammatory cells responsible for arthritic disease progression.

The noninvasive nature of this imaging technique could make it valuable for translation into the clinic, especially since proper monitoring of RA could greatly impact the diagnostic and treatment phases for patients with arthritis and could even help assess treatment response [128]. It may be worth exploring other possible target markers present in inflamed arthritic tissue (such as VCAM-1) [138].

### 5.4. αSyn: Tracking Neurodegenerative Disorders

α-Synuclein (αSyn) is a prion-like protein whose aggregation is known to dictate the development of many neurodegenerative diseases, including Parkinson’s, dementia with Lewy bodies [139], PD dementia, multiple system atrophy, and other related synucleinopathies [140]. While its exact mechanism of action still remains a mystery, monitoring αSyn could prove essential to better understanding these common diseases and detecting them in their early and controllable stages.

Two different nanobodies were developed to bind to the accessible C-terminal domain of αSyn [141,142]. Via single-molecule fluorescence, both were found not only to effectively inhibit αSyn fibril formation but also to decrease the stability of the αSyn oligomers upon binding. This suggested at the possible therapeutic potential of these anti-αSyn nanobodies, as their binding triggered significant decreases in oligomer-related cellular toxicity [141]. The nanobodies could also be imaged without first being radiolabeled, as they were found to be cross-reactive to Rpn10, a proteasomal subunit. This means that although the nanobody is continually expressed, even in the absence of αSyn, it would be degraded by the proteasome unless it could be stabilized through binding to αSyn [140]. The natural clearance of the anti-αSyn nanobodies from cells allowed for a fluorescence imaging technique, by fusing the nanobodies to fluorescence proteins that would provide varying signals based on the presence or absence of intracellular αSyn. Tracking intracellular αSyn has many implications, as cellular uptake of toxic αSyn proteins is thought to play an important role in αSyn transmission and neurological disease progression. This Fluorescent Reporter for human αSyn (FluoReSyn) therefore holds potential for translation into the clinic, as it provides a means to track the localization of transmittable αSyn aggregates in the cerebrospinal fluid [140,143]. If implemented in the clinic, using FluoReSyn in conjunction with these anti-αSyn nanobodies may better inform the diagnoses and treatment plans of neurodegenerative disorders that would otherwise be too elementary to detect, potentially increasing the life expectancies of patients.

### 5.5. DPP6: Imaging Insulin-Secreting Cells in Diabetes

The loss of beta cells is characteristic of type I diabetes and often seen in patients who have recently undergone islet transplantation procedures. However, because their process of depletion in such diseases is frequently variable and unpredictable, beta cells and endocrine cell masses are difficult to track and quantify in vivo without pre-labeling the islet cells prior to implantation or modifying them post-injection [144,145,146]. This difficulty stems from the fact that endocrine cells only contribute around 1–2% of the total pancreatic mass and also are not conveniently concentrated in a predictable, single area of the pancreas [146]. Developing a reliable way to target and specifically image alpha and beta cells, therefore, could help offer a greater understanding of the mechanism behind beta cell loss in diabetic patients and provide a streamlined way of evaluating the efficacy of treatments and medications.

A systems biological approach and RNA sequencing of pancreatic islets helped identify dipeptidyl-peptidase 6 (DPP6) as a highly specific pancreatic biomarker for diabetes and insulinomas [145]. The mRNA expression of DPP6 was found to be 25-fold higher in human pancreas islet cells than in neighboring tissues, and several-fold higher than any other tissues throughout the body except the brain, where it was comparable. Immunohistochemistry of human pancreas using commercial anti-DPP6 mAbs found DPP6 immunoreactivity in 90 ± 3% of insulin cells and 74 ± 10% of glucagon cells (n = 3), with little to no DPP6-positive cells in the exocrine pancreas. This specific expression of DPP6 was further reinforced by the fact that it, following translation to the protein level, demonstrated accumulation in only the alpha and beta islet cells of the pancreas. This is advantageous, as tracking alpha cells is also important because beta cells are known to often degranulate or differentiate and result in the formation of hormone-negative cells [147,148]. Not only this, but alpha cells can also conveniently be trans-differentiated into beta cells [149]. The DPP6 expression observed in human islet cells was not modified by the presence of pro-inflammatory cytokines, such as IL-1ß and IFN-γ, suggesting that the inflammatory responses prevalent in type I diabetes would not affect expression of the biomarker during imaging [145]. DPP6 demonstrates very limited expression in exocrine cells and extra-pancreatic tissues. Therefore, DPP6 satisfies many of the criteria necessary to be a promising target for radiotracers and imaging probes.

An anti-DPP6 nanobody was developed, radiolabeled with ^99m^Tc, and used for SPECT/CT imaging of immunodeficient mice transplanted intramuscularly with DPP6-expressing neuroblastoma cells or insulin-producing human EndoC-ßH1 cells [145]. The ^99m^Tc-anti-DPP6 nanobody displayed a two times higher uptake in the neuroblastoma tumor (1.2 ± 0.10% IA/g) than in negative control mice (0.5 ± 0.1% IA/g). This specific uptake was also seen for the EndoC-ßH1 tumor (1.0 ± 0.1% IA/g) in comparison to the negative control mice (0.5 ± 0.04% IA/g). Not only was the anti-DPP6 nanobody taken up specifically in areas of interest, but it also demonstrated rapid clearance from nontargeted tumors and blood. Flow cytometric analysis confirmed the nanobody’s ability to bind to human endocrine tissue as well as inability to recognize nonspecific exocrine tissue. Radioactivity signals from the ^99m^Tc-anti-DPP6 nanobody also showed notably high tumor-to-blood (2.9 ± 0.2 and 2.5 ± 0.4 in mice with neuroblastoma and mice with EndoC-ßH1, respectively) and tumor-to-muscle ratios (9.9 ± 3.2 and 9.9 ± 2.2, respectively). This allowed for clear, high-contrast nanobody visualization in the generated SPECT/CT images. A recent study used a ^99m^Tc-labelled anti-DPP6 nanobody and performed imaging on severe combined immunodeficient (SCID) mice transplanted with EndoC-βH1 cells, human islets, or pancreatic exocrine tissue. Results showed that the DPP6 protein is expressed mainly in pancreatic islets and that the nanobody can detect high amounts of EndoC-βH1 cells or human islets grafted in SCID mice [150]. Translation of the ^99m^Tc-anti-DPP6 nanobody into the clinic could thus prove largely useful in quantifying endocrine cell masses, ultimately allowing for the noninvasive, in vivo monitoring of diabetic treatment progress and patient recovery from islet cell implantations.

### 5.6. KC: Imaging Liver Inflammation and Pathogenesis

The liver plays a well-known role in metabolism as well as the clearance of foreign pathogens and toxins from the blood. This, however, makes the liver vulnerable to a variety of diseases, namely those associated with metabolism, drug-induced toxicity, or hepatodestructive immune responses [151,152]. Currently, the stages of liver disease can only be reliably determined via invasive procedures, such as biopsies. Developing noninvasive methods to assess liver pathogenesis is thus an important step towards improving the diagnostic procedure of hepatological disease and monitoring the effectiveness of everyday treatment.

Kupffer cells (KCs) display many characteristics that have deemed them promising targets for monitoring liver inflammations and pathogenesis [153]. As liver resident macrophages, their primary role is to facilitate liver function by mediating tissue homeostasis and toxin clearance. Importantly, however, KCs respond dynamically to inflammatory stimuli and express unique biomarkers to signal hepatoprotective and hepatodestructive immune responses [151]. C-type lectin domain family 4 member F (Clec4F) and V-set and immunoglobulin domain containing 4 (Vsig4) are two recently recognized KC markers and have thus been studied for their potential use as KC imaging targets [151,154].

Two different nanobodies were generated against recombinant mouse Clec4F (gene name: C-Type Lectin Domain Family 4 Member F) and Vsig4 (gene name: V-Set and Immunoglobulin Domain Containing 4) and then radiolabeled with ^99m^Tc to visualize the KC dynamics using SPECT/µCT [151,154]. Unlike Clec4F, however, Vsig4 is not a marker exclusive to KCs, as it is also expressed on macrophages residing in the heart, adrenal glands, and peritoneal cavity during inflammation [155]. Since Clec4F is expressed specifically in mouse livers and in no other organs, the anti-Clec4F nanobody was used more extensively in this study to allow for maximal selectivity when tracing and targeting KCs [151,156]. Imaging was performed on mice with concanavalin A (ConA)-induced autoimmune hepatitis and mice with non-alcoholic steatohepatitis (NASH) to analyze the evolution of these liver pathogeneses. One hour following the tracer injection, tissue and biodistribution analyses demonstrated specific accumulation of the anti-Clec4F nanobody in the liver, kidney, and bladders, suggesting not only specific detection of the antigen in the liver, but also the rapid blood clearance of unbound probes. Ex vivo analysis with flow cytometry found that Clec4F was only expressed on CD11b^int^ F4/80^+^ KCs, as it colocalized with F4/80-expressing cells and was detected on KCs but not on monocytes or polymorphonuclear cells.

In mice with ConA-induced hepatitis, there was an observed decrease in radioactive signal from the anti-Clec4F nanobody, visible as early as 3 h and as late as 48 h post-injection [151]. Flow cytometry and immunohistochemistry confirmed the correlation of this weaker signal with the reduction in KC number in these mice. Recovery of normal signal and restoration of normal KC numbers occurred 72 h post-injection, which coincided with the resolution of the ConA-induced hepatitis condition. This correlation proves that the anti-Clec4F nanobody does indeed bind specifically to KCs in the liver, which could potentially make it a powerful tool for the imaging of liver pathogenesis.

To further explore the potency of these KC-specific nanobodies, mice with NASH were also injected with anti-Clec4F and anti-Vsig4 and imaged with SPECT/CT [151,157]. In contrast to the results from mice with ConA-induced hepatitis, however, steatohepatitis led to higher nanobody signals in the liver, corresponding to an increased density of KCs rather than a reduction [151]. Flow cytometry and immunohistochemistry indeed verified this increase in number of KCs/g of liver both in vivo and ex vivo, detected by both anti-Clec4F and anti-Vsig4 nanobodies. These differences in KC dynamics and numbers for the two liver diseases prove that the KC landscape in the liver is transiently modulated and receptive to environmental changes, showing that KCs are powerful imaging targets that can provide insight into liver disease.

In preclinical applications, anti-Clec4F and anti-Vsig4 can and have helped with scientific research in drug efficacy, drug toxicity, and disease monitoring [151]. However, while these nanobodies would prove beneficial in the clinic, it is important to note that, in humans, Clec4F is also expressed in the colon, brain, testis, and ovaries to nontrivial extents [158]. This is in contrast to its exclusive expression on KCs in mice, and thus may not be ideal for translation into the clinic. In that regard, the anti-Vsig4 nanobody shows slightly more promise. Although Vsig4 also is not an entirely KC-specific biomarker, the results of this study demonstrated that its effectiveness in imaging NASH and autoimmune hepatitis was not significantly reduced by this lack of specific expression [151,154]. This study encourages continuing the search for other, potentially more specific human KC biomarkers.

### 5.7. Summary of Imaging Non-Cancer Targets

Overall, nanobodies have demonstrated powerful potential in imaging not only various types of cancers, but also other diseases that are often difficult to track until their later stages. By monitoring the microenvironments of diseased regions, progression can be tracked in real-time, treatments can be better informed, and patient risks can be carefully assessed. Identifying the best possible markers to target has proven challenging for many non-cancer diseases, but ones have been found and used in successful imaging for fibroses, atherosclerosis, rheumatoid arthritis, and certain neurodegenerative, pancreatic, and hepatological diseases. While the vast majority of relevant studies are preclinical, many of them have promising potential for translation into the clinic due to the significant benefit it would offer in the realms of preventative care and patient monitoring.

## 6. Conclusions and Future Directions

Biopsies will likely remain the gold standard of cancer diagnostics for the foreseeable future; however, biopsies can sometimes be unrepresentative of the greater TME or targeted organ. Non-invasive immuno-PET imaging, as an adjunct to biopsies, can provide a holistic view of the TME and offer complete insight into both primary and metastatic tumors. Information revealed via imaging can help to make informed treatment decisions. Imaging is also beneficial in understanding the progression and pathogenesis of a variety of diseases, such as fibroses, cardiovascular complications, arthritis, and neurological diseases. Therefore, immuno-PET imaging is a potentially revolutionary addition to disease management and treatment.

The use of radiolabeled nanobodies as imaging probes overcomes many of the weaknesses of using full-size antibodies and larger antibody fragments. Nanobodies have excellent tissue penetration, rapid blood clearance profile, high specificity, low nanomolar to picomolar affinity for the target, high stability and water solubility, ease of production, and a pharmacokinetic profile compatible with short-lived radioisotopes. One major drawback of nanobody-PET is the high renal retention and toxicities, similar to other radiotherapeutics and imaging agents [159,160]; however, techniques such as PEGylation can help decrease kidney retention [43]. Other strategies to decrease renal toxicity involve the co-infusion of basic amino acids such as lysines, or the use of gelofusine, which is a gelatin-based plasma expander [161,162]. Gelofusine mediates a decrease in kidney uptake through the interference of its plasma expander with the tubular reabsorption of nanobodies. Immunogenicity has also been reported for some nanobodies, though it may be idiotypic and specific only to the variable regions. Once again, techniques such as PEGylation may help to decrease immunogenicity in addition to undesirable kidney retention [32,69]. A recent study assessed the immunogenicity risk profiles of two nanobodies –– anti-HER2 and anti-CD206 (MMR) –– that have advanced into Phase II clinical trials for PET imaging. Strikingly, only 1 patient out of 20 showed a minimum amount of pre-existing anti-VHH antibodies, which was only marginally increased several months post-injection of the nanobody. Assessing the in vitro immunogenicity of the nanobodies using human dendritic cells did not induce T cell activation, further suggesting a low immunogenicity profile of nanobodies [163].

These issues of kidney retention and immunogenicity require better understanding and further investigation, as overcoming them would contribute greatly to clinical success. Similar to other antibody-based imaging approaches, nanobody-based imaging agents are the most effective when selected epitopes demonstrate a few common characteristics. These include antigen recognition through expression on the extracellular surface of the plasma membrane, availability of the epitope for similar recognition, high expression of the antigen on the cell surface, and little to no expression in normal tissues.

Radiolabeled nanobodies can provide valuable information about the biological processes taking place inside living organisms, giving researchers and clinicians the appropriate data that are needed to improve patient care. For example, nanobodies can be used to understand the dynamic of immune responses, helping to gain mechanistic insight into how the tumor immune landscape is shaped and responds to treatment. Understanding the response mechanisms, in turn, may lead to the identification of new targets and avenues to pursue for developing new therapeutics or biomarkers. Several studies have been performed on imaging lymphocytes, checkpoint molecules, and cancer markers; the recent more in-depth understanding of the tumor immune landscape suggests myeloid cells play a central role in shaping the TME. Therefore, pursuing the development of novel nanobodies for imaging specific subsets of myeloid cells can turn out to be both important and advantageous. Similarly, as cytokines and chemokines are key players in the pathogenesis of disease, imaging their level of presence and movement may help to gain insight into understudied disease pathogeneses and progression models. Taken together, understanding the behavior of immune cells and immune-modulating molecules before, during, and after treatment will help to decide the best course of treatment for patients and allow for a dynamic and adaptable inpatient care experience. Harnessing the powerful imaging potential of nanobodies would be an ideal strategy to tackle these issues and ultimately achieve such ideal outcomes.

To ensure maximal effectiveness and minimal nonspecific binding, it could be worthwhile to continue searching for other targetable markers associated with the diseases mentioned in this review, but the major priority in coming years may better be focused to expand the number of different diseases that can be imaged and characterized by radiolabeled nanobodies, such as inflammation markers to diagnose fever of unknown origin, neurodegenerative disease such as Alzheimer, and cytokines, chemokines, and their receptors that are key in pathogenesis and progression of disease. By helping scientists better understand and visualize the driving forces behind disease progression, expanding the library of nanobody-based imaging agents will become an even more promising tool in guiding the development of novel, effective treatment plans for patients in the future.

The generation of nanobodies is now a well-established procedure [33,164]. The increasing availability of commercial sources for immunization and identification of lead candidates, along with advancements in development of synthetic libraries will continue to help provide easier access to new nanobodies against antigens of interest. While we have focused on the imaging applications of nanobodies, they also can be used as therapeutics, as a molecular biology tool for mechanistic studies, and to investigate biological processes. With the recent FDA approval of a nanobody-based treatment (Caplacizumab, a bivalent nanobody) and the clinical translation of several nanobodies, the repertoire of available nanobodies is only expected to grow in the years to come.

**Table 1:** Nanobodies developed for noninvasive immuno-PET/SPECT imaging.

Target	Agent	Reactivity	Clinical Trials: Stage and Status (If Applicable)	References
EGFR	^99m^Tc-8B6	Human	Preclinical	[38]
	^99m^Tc-7C12	Human	Preclinical	[48]
HER2	^177^Lu-2Rs15dHIS	Human	Preclinical	[55]
	^18^F-FB-2Rs15d	Murine	Preclinical	[56]
	^18^F-RL-I-5F7	Murine	Preclinical	[57]
	^68^Ga-2Rs15d	Human	Clinical	[56,165]
HER3	^89^Zr-MSB0010853	Murine	Preclinical	[62]
CEA	^99m^Tc-NbCEA5	Human	Preclinical	[65]
PSMA	^111^In-JVZ007	Human	Preclinical	[59]
HGF	^89^Zr-1E2, ^89^Zr-6E10	Human	Preclinical	[67]
CD20	^68^Ga-9079	Human	Preclinical	[70]
CD38	^68^Ga-NOTA-Nb1053	Murine	Preclinical	[60]
Mesothelin	^99m^Tc-A1, ^99m^Tc-C6	Human	Preclinical	[76]
MMR	^99m^Tc-d a-MMR Nb cl1	Murine	Preclinical	[128,166]
	^ 18 ^ F-FB-anti-MMR 3.49	Human, Murine	Preclinical	[123]
	^68^Ga-NOTA-Anti-MMR-VHH2	Human	Clinical, NCT04168528 (Active)	[124]
MHC II	[^18^F]FDG -VHH7	Murine	Preclinical	[117]
	^64^Cu- VHH4	Human	Preclinical	[121]
CD11b	^89^Zr-VHHDC13 (PEGylated)	Murine	Preclinical	[115]
	^18^F-VHHDC13	Human	Preclinical	[118]
CD8	^89^Zr-VHH-X118 (PEGylated)	Murine	Preclinical	[43]
	^68^Ga-NOTA-SNA006	Human	Preclinical	[119]
Mouse Dendritic Cells	^99m^Tc-Nb-DC2.1	Murine	Preclinical	[167]
	^99m^Tc-Nb-DC1.8	Murine	Preclinical	[167]
PD-L1	^18^F-B3, ^18^F-A12, ^64^Cu-B3	Murine	Preclinical	[95]
	^99m^Tc-C3, ^99m^Tc-C7, ^99m^Tc-E2, ^99m^Tc-E4, ^99m^Tc-K2	Murine	Preclinical	[92,93,94,168]
	^68^Ga-NOTA-Nb109	Human	Preclinical	[169]
	^99m^Tc-NM-01	Human	Clinical, NCT02978196 (Concluded)	[98]
	^89^Zr-envafolimab (Fc fusion)	Human	Clinical, NCT03638804 (Active)	[99,100]
CTLA-4	^18^F-H11, ^89^Zr-H11	Murine	Preclinical	[100,106]
LAG-3	^99m^Tc-anti-moLAG-3 3206, ^99m^Tc-anti-moLAG-3 3208, ^99m^Tc-anti-moLAG-3 3132, ^99m^Tc-anti-moLAG-3 3141	Murine	Preclinical	[110,111]
VCAM-1	^ 99m ^ Tc-cAbVCAM1-5	Human, Murine	Preclinical	[128,130,170,171]
FN-EIIIB (ECM)	^ 64 ^ Cu-NJB2	Human, Murine	Preclinical	[80]
αSyn	NbSyn2, NbSyn87 (fused to fluorescent proteins for imaging)	Human	Preclinical	[140,141]
DPP6	^ 99m ^ Tc-4hD29	Human	Preclinical	[145]
Vsig4	^ 99m ^ Tc-NbV4	Murine	Preclinical	[151,154]
Clec4F (KC)	^ 99m ^ Tc-NbC4	Murine	Preclinical	[151]

## Data Availability

Not applicable.
